# Myxedema coma secondary to levothyroxine malabsorption in a patient previously submitted to bariatric surgery

**DOI:** 10.20945/2359-4292-2023-0095

**Published:** 2024-02-19

**Authors:** Caterina Buoso, Maria Cavadini, Paolo Facondo, Valentina Anelli, Virginia Maltese, Francesca Bambini, Elisa Gatta, Andrea Delbarba, Carlo Cappelli, Ilenia Pirola

**Affiliations:** 1 University of Brescia Department of Clinical and Experimental Sciences Brescia Italy Department of Clinical and Experimental Sciences, SSD Endocrinologia, University of Brescia, Azienda Socio-Sanitaria Territoriale (ASST) Spedali Civili di Brescia, Brescia, Italy

## Abstract

Treating hypothyroidism can be challenging in patients with malabsorption, as they require a higher daily dose of oral levothyroxine (L-T4). Oral L-T4 absorption occurs mainly in the jejunum and the ileum and is affected by gastric acidity. As a result, absorption can be impaired by bariatric surgery. This paper presents a case of myxedema in a young man who had previously undergone biliopancreatic diversion. He was referred to the Emergency Department with deteriorated mental state, hypotension, bradycardia and hypothermia. Laboratory tests revealed severe hypothyroidism and hypokalaemia. The clinical and biochemical profile of the patient suggested myxedema coma. The tablet-based L-T4 therapy was replaced with intravenous (iv) L-T4, oral liquid L-T4 and oral liothyronine (L-T3) and inotropic agents and supportive care were also administered, resulting in a gradual improvement in clinical condition. The patient reported taking L-T4 tablets as prescribed before hospitalization. In patients with malabsorption, impaired L-T4 absorption may lead to severe forms of hypothyroidism. This case outlines the need for more frequent monitoring of serum Thyroid Stimulating Hormone in patients submitted to bariatric surgery and suggests the benefit of using liquid L-T4 in the place of tablets in cases of malabsorption.

## INTRODUCTION

Community surveys reveal that hypothyroidism affects up to 5% of the general population, with a further 5% going undiagnosed (1–7). All major Endocrine Societies’ recent guidelines recommend L-T4 monotherapy as first-line treatment for patients with hypothyroidism (8,9). L-T4 absorption occurs mainly in the jejunum and the ileum and is optimal with an empty stomach, which demonstrates the fact that gastric acidity plays a key role in this process. In fact, it is well known that food and drugs can reduce the bioavailability of L-T4, at least for tablets (10). Therefore, L-T4 tablets must be taken on an empty stomach, at least one hour before breakfast or before bed. For patients consuming a breakfast of similar daily food choices and avoiding foods known to interfere with LT4 absorption, it is also reasonable to take L-T4 tablets even 30 minutes before eating (9). In addition, various pathological conditions can impair L-T4 absorption and it is common for higher daily doses of L-T4 to be prescribed for patients who have had jejunoileal bypass surgery or experienced bowel resection after surgery (10).

Myxedema is a rare, life-threatening complication of untreated or undetected hypothyroidism. It is an endocrine emergency that involves multiple organs and may require hemodynamic, respiratory and neurological support in addition to thyroid hormone replacement therapy (11).

Obesity has reached epidemic proportions worldwide. According to the National Health and Nutrition Examination Survey 2017-March 2020, the prevalence of obesity in adults in the United States is 41.9% (12). Simultaneously, the number of bariatric surgeries being performed has significantly increased in recent years, reaching 200,000 procedures in the United States in 2020 (13). Surgical techniques that induce malabsorption of nutrients (e.g., biliopancreatic diversion) are more likely to lead to drug absorption issues. The primary mechanisms that explain this are: decreased absorptive surface area and acidic environment (14). Oral L-T4 absorption is significantly influenced by gastric acidity and therefore can be affected by bariatric surgery (15–17).

Nowadays, no cases of myxedema coma in patients previously submitted to bariatric surgery are reported. We recounted a life-threatening case of myxedema in a young, obese man who had previously undergone biliopancreatic diversion surgery.

## CASE REPORT

In November 2022, a 47-year-old man was referred to our Emergency Department for the deterioration of his mental state, cognitive-motor slowing and apathy. He also complained of abdominal pain and general weakness. His medical history included hypothyroidism due to autoimmune thyroiditis on stable L-T4 therapy for 15 years, past alcohol and cocaine abuse as well as bariatric surgery. He had undergone biliopancreatic diversion 10 years earlier with a BMI of 40.2 kg/m^2^ and a consequent wight loss of more than 30 kg (current BMI 29.5 kg/m^2^).

At admission, the patient was hypotensive (95/50 mmHg), bradycardic (35 beats/min) and hypothermic (33 °C). Upon physical examination, his face appeared jaundiced and puffy, with non-pitting oedema in his lower extremities. Upon examining his abdomen, the patient reported diffuse tenderness.

**Figure 1:**
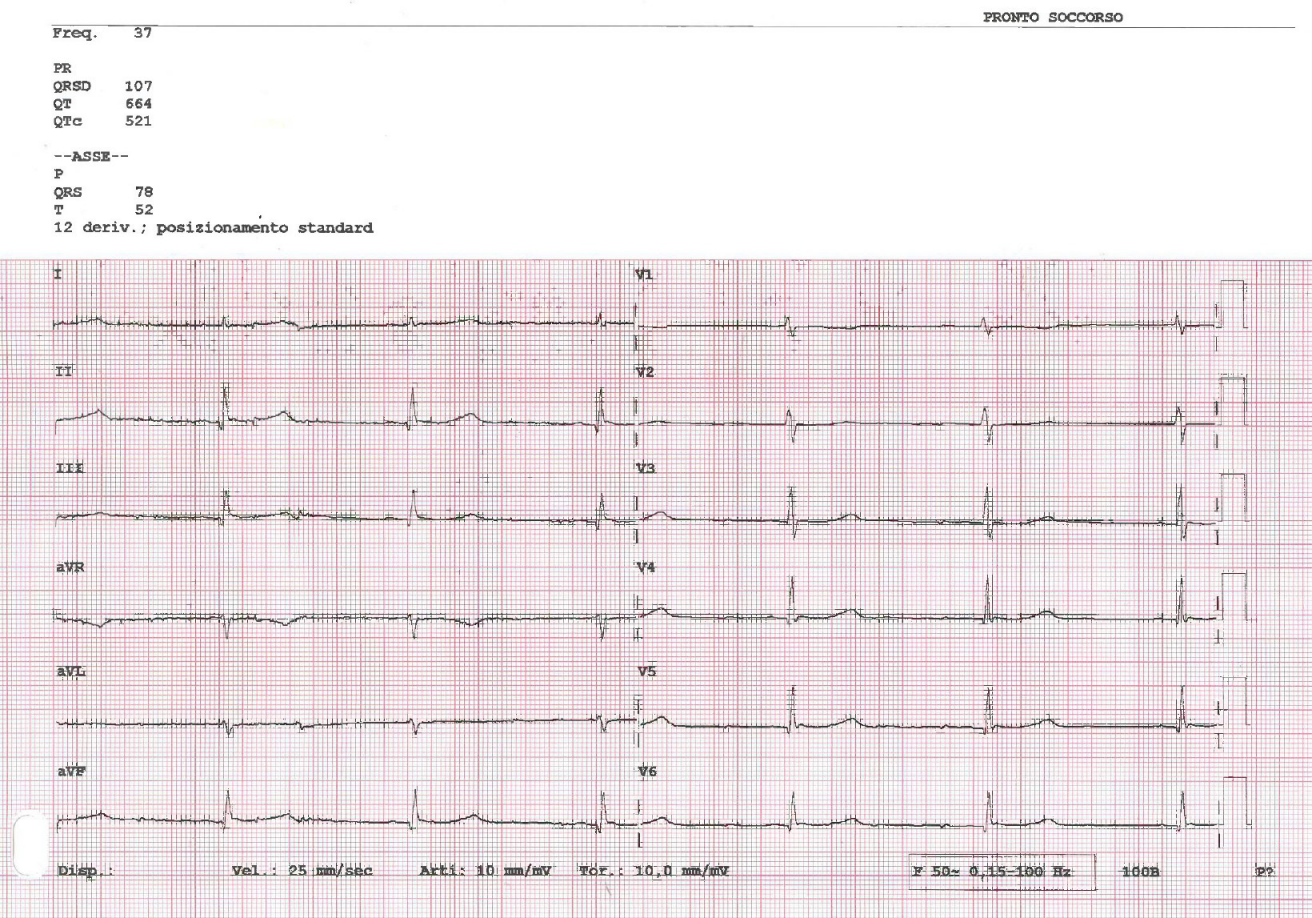
ECG at presentation showing marked bradycardia (37 beats per minute) and prolonged QTc (520 msec).

The laboratory tests showed severe hypothyroidism, with a Thyroid Stimulating Hormone (TSH) of 206 mIU/L (normal range 0.27-4.20 mIU/L) and a free Thyroxine (fT4) of 2.43 ng/mL (normal range 9.30-17.00 ng/mL), and hypokalaemia, with a serum potassium level of 2.7 mmol/L. Both blood alcohol levels and urinary cocaine metabolites were negative. ECG showed prolonged QTc of 520 msec and marked bradycardia (Figure 1), while echocardiogram reported preserved left ventricular function and kinetics (Ejection Fraction = 60%). The clinical and biochemical profile suggested myxedema coma.

The patient was admitted to our Internal Medicine Unit and was placed under continuous monitoring of vital parameters. Blood tests confirmed severe hypothyroidism; the results showed macrocytic anaemia, increased levels of creatinine, hypokalaemia, hyperlipaemia, suggesting concomitant pancreatitis (Table 1). A Computed Tomography (CT) scan of the chest and abdomen revealed acute pancreatitis.

**Table 1:** Laboratory findings at admission showing severe hypothyroidism, hypokalaemia, macrocytic anaemia and hyperlipaemia

Laboratory values	Measured	Normal range
White blood cell count	7.58 × 10^3/uL	4.00-10.80
Red blood cell	3.14 × 10^6/uL	4.5-5.50
Hemoglobin (Hb)	10.3 g/dL	14.0-18.0
Hematocrit (HCT)	29.7 %	42.0-52.0
Mean cell volume (MCV)	94.6 fL	82.0-94.0
Platelets (PLT)	96 × 10^3/uL	130-400
Serum sodium level	137 mmol/L	136-145
Serum potassium level	2.7 mmol/L	3.4-4.5
Serum chloride level	109 mmol/L	98-107
Serum creatinine level	1.37 mg/Dl	0.70-1.20
Serum glucose	117 mg/dL	78-106
Serum calcium level	7.46 mg/dL	8.60-10.20
Serum magnesium level	1.01 mmol/L	0.66-1.07
Serum phosphorous level	3.1 mg/dL	2.8-4.8
Serum albumin level	27.97 g/L	31.00-52.00
Aspartate aminotransferase	22 U/L	10.0-50.0
Alanine aminotransferase	17 UL	10.0-17.0
Gamma-glutamyltransferase	115 U/L	50-116
Lipase	2357 U/L	13-60
C-reactive protein	14.5 mg/L	<5.0
Thyroid stimulating hormone	206 mIU/L	0.270-4.200
Free thyroxine (fT4)	2.43 ng/mL	9.30-17.00
Ferritin	154 ug/L	30-400
Vitamin B12	564 ng/L	197-771

The patient was immediately administered 42 °C saline solution at 250 mL/h, as well as hydrocortisone 100 mg twice a day, intravenously. Oral liquid L-T4 100 mcg and L-T3 6 drops three times daily were initiated. In addition, L-T4 400 mcg intravenous was administered for the first two days. Given the persistence of bradycardia and hypotension, our intensive care specialists prescribed isoprenaline 1 mg in saline solution 50 mL at 0.03 mL/h until heart rate reached > 50 bpm. Hypokalaemia was corrected with glucose solution 10% added with KCI 10 mEq at 42 mL/h. On the second day, intravenous L-T4 was stopped. Oral thyroid hormone replacement therapy was gradually increased, reaching a dose of 28 drops three times a day of L-T3 and 150 mcg of L-T4 daily. Hydrocortisone was gradually reduced and stopped. Isoprenaline 1 mg in saline solution 50 mL at 0.03 mL/h and theophylline 200 mg twice a day were also administered in order to maintain a heart rate of over 60 bpm for another 2-3 days. L-T3 was gradually withdrawn and only liquid L-T4 continued to be administered. After five days of therapy, thyroid function gradually improved (fT4 23.4 and fT3 4.3), as well as vital parameters (blood pressure: 120/70 mmHg, heart rate: 42 bpm and body temperature: 36.7 °C) and neurological status. The patient, when questioned about the correct L-T4 replacement therapy intake, confirmed that he followed the daily prescription (tablet formulation), but he was unsure of the dosage (100 mcg or 125 mcg). He admitted that he had assumed L-T4 in liquid formulation for the first nine years and then switched to tablets for convenience. According to clinical records, the patient’s serum TSH level was in the normal range in October 2021 (*i.e.*, in L-T4 liquid formulation) and 44 mIU/L in July 2022 (i.e. in L-T4 tablet formulation). He also assumed vitamin D daily and iron for the first week every month, both taken in the afternoon several hours after L-T4 assumption. No diarrhoea or other alteration in bowel habits in the months preceding the admission were reported.

When discharged, the patient’s myxedema had improved, and he was prescribed continuing therapy of L-T4 liquid formulation (150 mcg/die). He was referred to the Endocrinology Unit in order to manage the hypothyroidism until stable euthyroidism is achieved.

## DISCUSSION

Myxedema coma is a rare but potentially lethal complication of hypothyroidism (18). The syndrome occurs almost exclusively in older patients (>60 years of age) and may be determined by multiple precipitating factors, including: exposure to the cold, infections, trauma or anaesthesia. More than 90% of cases occur in women during the winter months (19,20). In addition, myxedema coma has not been classified following long-term withdrawal from replacement therapy, among patients after surgical resection for thyroid cancer or patients who have undergone radio-ablative treatment (21). Clinical presentation is typically characterized by a deterioration in mental state and hypothermia, but bradycardia, hypoglycaemia and kidney and respiratory failure are often present (11). The treatment of myxedema is a multisystem challenge and requires the continuous monitoring of vital parameters (22). Thyroid hormone replacement therapy (L-T4 and L-T3) is mandatory, and according to the American Thyroid Association (ATA) guidelines, the recommended initial dose of LT4 is 200 to 400 mcg iv. Moreover, it is advised to administer hydrocortisone prior to thyroid hormone therapy especially if the patient is hypotensive, in order to prevent an adrenal crisis (9). If hypotension is refractory to iv fluid resuscitation, vasopressors should be initiated, as well as inotropic support in the case of heavy bradycardia. Furthermore, hypothermia can be treated using heated blankets and/or heated saline solution iv.

To the best of our knowledge, few cases of myxedema coma due to L-T4 malabsorption have been reported in literature. Wiggins and cols. recounted a case of a 55-year-old male patient on long-term L-T4 replacement therapy newly diagnosed with coeliac disease. A restrictive coeliac diet resolved L-T4 malabsorption and the concomitant myxedema (23). In addition, Ahmad and cols. reported the case of a 30-year-old woman affected by polyglandular autoimmune type 2 syndrome (primary autoimmune hypothyroidism, primary autoimmune adrenal insufficiency, type 1 diabetes mellitus, coeliac disease), unable to adhere to a gluten-free diet (24). A very recent study by Mc Donald and cols. reported a case of immune-related enteritis malabsorption in a female patient on treatment with immune checkpoint inhibitors (25). Reardon and cols. reported a case of a patient with gastroparesis secondary to type I diabetes mellitus. The malabsorption, and consequently the myxedema, was resolved by L-T4 liquid formulation in place of tablets (26).

We recount the first case of myxedema coma in a young patient who had previously undergone bariatric surgery. Our patient, questioned several times, reported regular assumption of L-T4 at home according to prescription before hospitalisation, even if he was unsure of the dosage (100 *vs.* 125 mcg/daily). He reported taking L-T4 in liquid formulation for the first nine years of therapy: in accordance with his medical record, serum TSH was in the normal range during therapy with liquid formulation. In addition, the patient admitted that he had switched to tablets one year earlier for convenience: the last TSH value available whilst on tablet formulation, four months before hospitalization, showed evidence of hypothyroidism. No precipitating factors were identified, except for the preceding malabsorptive bariatric treatment. This procedure could also determine drug malabsorption "per se" (14). It is well-known that most drugs, including levothyroxine, are mainly absorbed in the small intestine, which is bypassed in biliopancreatic diversion (27,28). New liquid formulation seems to overcome this problem as clearly shown in the different set-points (10,29,30). Notably, our group showed TSH normalization in four patients who underwent Roux-en-Y gastric bypass surgery after switching to liquid L-T4 formulation (15). The same result was also obtained by Fallahi and cols. in 17 patients, 13 who underwent Roux-en-Y and four who underwent biliary pancreatic diversion (31). In addition, the effectiveness of the liquid formulation has already been demonstrated in other malabsorption conditions, such as Helicobacter pylori infection (32) or atrophic gastritis (33). For this reason, liquid formulations should be preferred in non-compliant patients and in any condition that could impair L-T4 absorption (10,34).

In conclusion, we reported the first case of myxedema coma in a young patient who had previously undergone bariatric surgery. The procedure, which reduces L-T4 tablet absorption, can lead to severe forms of hypothyroidism. We suggest checking serum TSH more frequently in this group of patients. Finally, liquid L-T4 formulation should be an option for all patients with malabsorption.
